# A Physical Rehabilitation Approach for Parkinson’s Disease: A Systematic Literature Review

**DOI:** 10.7759/cureus.44739

**Published:** 2023-09-05

**Authors:** Andrea Tobar, Arturo P Jaramillo, Stefany C Costa, Karla T Costa, Sandy S Garcia

**Affiliations:** 1 Rehabilitation Medicine, Pontificia Universidad Católica del Ecuador (PUCE), Quito, ECU; 2 General Practice, Universidad Estatal de Guayaquil, Machala, ECU; 3 General Medicine, Pontificia Universidad Católica del Ecuador (PUCE), Quito, ECU; 4 Medicine, Pontificia Universidad Católica del Ecuador (PUCE), Quito, ECU; 5 Internal Medicine, Hospital de los Valles, Quito, ECU

**Keywords:** parkinson's disease, exercise training, ataxic gait, resting tremor, physiotherapy education

## Abstract

Parkinson's disease (PD) is one of the most common neurological ailments. With diverse motor affectations (postural instability, resting tremor, bradykinesia, and rigidity), people with Parkinson's disease (PwP) have a broad spectrum of non-motor symptoms. These include autonomic function changes, cognitive deterioration, neuropsychiatric difficulties, and sleep interruptions. Psychological disturbances, such as anxiety and sadness, are common among PwP. This discomfort is often accompanied by a decrease in general functioning, both at work and in social contacts. Furthermore, people who are experiencing psychological distress have a quick decrease in both physical and cognitive capacities. Furthermore, Pwp who also suffer from anxiety and depression are more likely to acquire dementia. It is worth noting that studies have shown good outcomes in the treatment of physical disabilities in PWP and the various therapeutics available for each affected body part, such as in the legs when they have walking problems, resting tremor in their hands, or micrography, which is a common symptom in these patients. The medical research databases PubMed/Medline, Google Scholar, and the Cochrane Library were used to look for relevant materials. Upon meticulous scrutiny, a thorough investigation was conducted on the papers at hand. A total of 10 publications were meticulously selected based on stringent qualifying criteria. The present investigation examines various perspectives regarding the physical rehabilitation of individuals diagnosed with PD. The majority of therapeutic interventions employed revolve around cutting-edge technologies, such as virtual reality (VR), combined with exercise regimens. These interventions have demonstrated notable statistical significance in terms of enhancing various physical aspects, including endurance, performance, gait capacity, perception, and overall independence in daily life activities. One of the gathered studies makes use of the therapeutic benefits of yoga to help PwP deal with their anxiety and improve their mental health. Based on the aforementioned information, further investigation is required to ascertain the optimal approach for physical rehabilitation management and develop diverse strategies aimed at assisting individuals with PD in attaining physical autonomy.

## Introduction and background

The cerebral basal ganglia malfunction in Parkinson's disease (PD), a neurological illness that results from degenerative changes in the nervous system [1]. According to one source, it is fairly unusual for patients to struggle with postural control and mobility [2]. Gait freezing, a prevalent occurrence in the latter stages of PD, is a common symptom among sufferers. Despite the patient's best efforts to ambulate, there is a considerable slowing in lower-extremity advancement, increasing the likelihood of inadvertent tumbles and complicating the patient's treatment regimen [3,4]. Currently, drugs aimed at addressing gait freezing in PD do not give an entirely efficient response to the individual [5]. Based on the study's results, it has been proposed that physical therapy might possibly help improve motor function in people with Parkinson's disease (PwP) [6,7].

One of the most promising therapies is virtual reality (VR), which showed a good outcome in the gait performance of PwP by providing visual, aural, and tactile stimulations. Users may engage with the VR environment while being watched and assessed by health experts. External stimuli have been shown to improve gait in PwP, with an added benefit in terms of enhanced velocity when visual cues are used [8,9]. Nonetheless, existing data are inadequate to support the assertion that VR technology improves motor function in PwP. The fast advancement of artificial intelligence has resulted in the establishment of innovative study fields in the field of rehabilitative medicine. Wearable sensors have the potential to give precise and impartial measures of balance exercises in PwP, according to new research [10]. The use of VR technology has grown in popularity as a novel approach to rehabilitation, and the body of literature on this topic has grown significantly in recent years. VR has the potential to provide patients with improved sensory input, producing a highly immersive experience, according to studies undertaken by specialists in the area. It also provides real-time feedback during certain motor actions. This has been shown to have a good effect on motor learning and neuroplasticity. Multiple sources back up these conclusions [10]. As a result, this strategy may be regarded as a supplement to traditional rehabilitation treatment. A pioneering meta-analysis revealed that "exergaming" has the potential to considerably enhance balance and functional mobility in healthy older people [10]. Expert studies have shown that visual information has the ability to act as a replacement for functional gait in circumstances when it may be absent [10]. According to the study, "exergaming" as a supported practice using the Kinect sensor is a safe and attractive replacement for traditional physical therapy for PwP [10].

The goal of this systematic literature review (SLR) is to examine the impact of various physical rehabilitation therapies on individual patients, taking into account the variety of approaches used by clinicians and the range of tools at their disposal. We have examined statistically significant findings in many areas, including the patient's sleep, the quality of their daily life duties on a mental-psychological level, and their physical capacity to execute things they previously found challenging.

## Review

Methodology

We conducted a systematic evaluation using free full-length papers and the Preferred Reporting Items for Systematic Reviews and Meta-Analysis (PRISMA) to describe our approach and results. 

Study Duration

This review started on May 24, 2023 and ended on July 25, 2023.

Search Strategy 

The search strategy is described in* *Table 1:

**Table 1:** Database searches from PubMed, the Cochrane Library, and Google Scholar

Search strategy	Databases used	Number of papers identified
Parkinson's disease AND tremor AND ataxia	The Cochrane Library	1,132
(( "Parkinson Disease/diet therapy"[Majr] OR "Parkinson Disease/mortality"[Majr] OR "Parkinson Disease/prevention and control"[Majr] OR "Parkinson Disease/psychology"[Majr] OR "Parkinson Disease/rehabilitation"[Majr] OR "Parkinson Disease/therapy"[Majr] )) AND (( "Tremor/diagnosis"[Mesh] OR "Tremor/prevention and control"[Mesh] OR "Tremor/psychology"[Mesh] OR "Tremor/rehabilitation"[Mesh] OR "Tremor/therapy"[Mesh] )) AND (( "Gait Ataxia/etiology"[Majr] OR "Gait Ataxia/prevention and control"[Majr] OR "Gait Ataxia/psychology"[Majr] OR "Gait Ataxia/rehabilitation"[Majr] OR "Gait Ataxia/therapy"[Majr] )) AND ( "Gait Ataxia/etiology"[Majr:NoExp] OR "Gait Ataxia/prevention and control"[Majr:NoExp]	PubMed	4,066
"Parkinson Disease[tw]" AND "Tremor[tiab]" AND "Ataxia[all]"	Google Scholar	444,000

Eligibility Criteria and Study Selection

In an effort to rigorously assess the eligibility of potential articles for inclusion in this medical systematic review, a pair of investigators conducted an in-depth review of each article's full title and content. Our methodological focus zeroed in on up-to-date literature, explicitly targeting articles published within the most recent five-year period from 2018 to 2023. Furthermore, language restrictions were imposed; only articles written in English or those having a freely accessible full-text English translation were considered for inclusion. If the full text of an article was inaccessible, the article was categorically excluded from the review. As a part of our stringent inclusion criteria, we carefully selected articles that explicitly address articles focusing on physical rehabilitation in PwP.

To maintain a high caliber of scientific rigor, gray literature and proposal papers were intentionally left out of the review. Through this multilayered, systematic approach to article selection, we aim to bolster the methodological integrity of our reviews. This, in turn, enhances its applicability and relevance in guiding both future research and evidence-based clinical practice in the field. In addition, for quality assessment, we used the Cochrane risk-of-bias assessment tools for randomized clinical trials (RCTs). 

Data Management

For the methodology of this medical systematic review, an evaluation process was employed to meticulously assess the eligibility and quality of potential articles for inclusion. Initially, two independent authors conducted a critical review of each article based solely on its title and abstract. Subsequently, abstracts deemed pertinent underwent a more detailed scrutiny through a complete, freely accessible full-text review. In instances where discord arose between the two initial reviewers, a third independent author was enlisted to perform an additional evaluation of the article in question. This triangulated approach was undertaken to mitigate bias and ensure a consensus in the selection process. Upon finalizing the roster of chosen studies, data extraction was carried out, targeting specific information for analytical priority. These data points included the first author's name, article type, year of publication, research design, and key results. Lastly, a thorough review for duplicate entries was performed to ensure the uniqueness and individual contribution of each article to the review. Any duplicates identified were systematically removed, thus maintaining the integrity and comprehensiveness of the research compilation.

Table 2 presents an in-depth description of the articles we decided to use.

**Table 2:** Data extraction results RCT, randomized clinical trial; BBS, Berg Balance Scale; TUGT, Timed Up and Go Test; FGA, Functional Gait Assessment; UPDRS3, Third Part of Unified Parkinson’s Disease Rating Scale; PwP, people with Parkinson's disease; PD, Parkinson's disease; CDTT, cognitive dual-task gait training; MDTT, motor dual-task gait training; SRTE, stretching and resistance training exercise; G-EO System: end-effector robotic device; RAS, rhythmic auditory stimulation; non-RAS, without RAS; CI, confidence interval

Author	Year of publication	Study design	Quality tool	Primary research	Outcome evaluation
Feng et al. [10]	2019	RCT	Cochrane risk-of-bias assessment tool	A total of 28 people with PD were assigned to two groups: the control group, which included 14 people, and the experimental group, which also included 14 people.	Following the intervention, both groups' FGA, TUGT, and BBS scores showed a P-value of 0.05. Nonetheless, when comparing the post-rehabilitation and pre-rehabilitation data of the placebo group, no significant difference in UPDRS3 scores was identified (p > 0.05). When compared to the standard physical therapy cohort, the use of VR training produced significantly better results (p > 0.05).
Pohl et al. [11]	2020	RCT	Cochrane risk-of-bias assessment tool	A total of 46 people with PD were randomly selected for the placebo group and treatment group. The placebo group received no instruction, and the treatment group received training incorporating a music-based intervention.	According to the reported outcomes, the music-based group seems to deliver considerable advantages in terms of mood improvement, alertness, and quality of life for PwP.
Amara et al. [12]	2020	RCT	Cochrane risk-of-bias assessment tool	Individuals with PD who were 45 years old and not engaged in a consistent exercise regimen were assigned to a control group (N = 28) or an exercise group that focused on sleep hygiene without any exercise (N = 27).	High-intensity exercise rehabilitation has been shown to improve objective sleep outcomes in PD patients. Exercise has been shown to be a very effective non-pharmacological strategy for improving this incapacitating non-motor symptom in PwP.
Yang et al. [13]	2019	RCT	Cochrane risk-of-bias assessment tool	Eighteen PwP were randomly allocated to either the MDTT, CDTT, or general gait training group.	Results showed a 0.3% decrease in mobility during the engaged cognitive task after CDTT. This decline was significantly greater than MDTT, which only showed a decrease of p = 0.006, and control training, which showed a decrease of p = 0.041.
Kwok et al. [14]	2019	RCT	Cochrane risk-of-bias assessment tool	Convenience sampling was used to recruit a total of 187 people, all of whom had idiopathic PD and were capable of standing alone and walking with or without help. These people were all at least 18 years old.	The findings revealed that the yoga group outperformed the SRTE group considerably. This was most noticeable in the categories of anxiety (p = 0.001), sadness (p = 0.001), and perceived equanimity (p = 0.001).
Cikajlo et al. [15]	2019	RCT	Cochrane risk-of-bias assessment tool	A total of 97 individuals were recruited for this extraordinary research, but owing to various limitations, only 20 suitable volunteers were chosen and randomly allocated to two unique groups. The first group got to try out the cutting-edge 3D Oculus Rift CV1, while the second had to make do with a standard laptop. The 10-session, three-week training program was attended by both sides.	The group using the 3D Oculus Rift CV1 had a spectacular and upstanding outcome in the average manipulation time when compared to group x time, with a statistically significant result (p = 0.009).
Chivers et al. [16]	2019	RCT	Cochrane risk-of-bias assessment tool	Individuals who are prone to falls and have PD were recruited for the research, which included specified subgroup analyses.	Secondary subgroup analysis revealed that the intervention had a different effect on the mild-to-severe disease severity cohorts. Chair stand time, fall efficacy, and balance improved significantly in the intervention arm, resulting in a substantial decrease in near falls.
Capecci et al. [17]	2019	RCT	Cochrane risk-of-bias assessment tool	The research entailed randomly assigning outpatients with PD to either 20 sessions of 45-minute walking training with the use of the treadmill training system or G-EO System.	The use of treadmill training and robot-assisted gait training resulted in increases in endurance and walking capacity. Furthermore, there was a 17% and 15% improvement in the quality of life and motor symptoms, respectively.
Calabro et al. [18]	2019	RCT	Cochrane risk-of-bias assessment tool	The research involved 50 people who were diagnosed with PD. Over the course of an eight-week training program, participants in this research were put into two separate categories of GaitTrainer3 equipment used during treadmill gait training.	Following RAS training, significant improvements were found in the PD scale rating (p = 0.00), the efficacy of fall scale (p = 0.001), and gait quality assessment (p = 0.001).
Ashburn et al. [19]	2019	RCT	Cochrane risk-of-bias assessment tool	A pragmatic masked investigator performed a multicenter, individually assessed RCT, including subgroup analyses.	The study's findings revealed no significant difference in the occurrence of repeated falls within six months of randomization (the OR between the control group and the PDSAFE group was 1.21, with a 95% CI ranging from 0.74 to 1.98; the p-value was 0.447).

Results

Search Results

**Figure 1:**
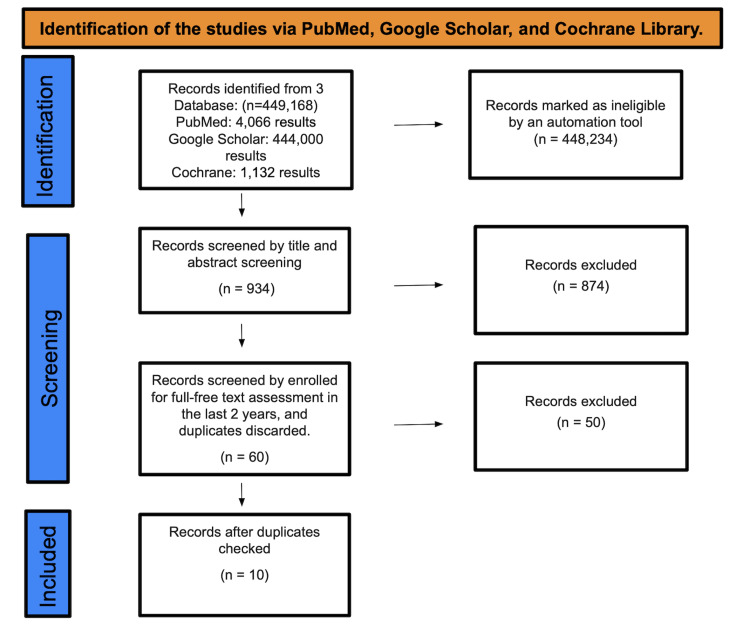
Identification of studies via databases and registers Preferred Reporting Items for Systematic Reviews and Meta-Analyses (PRISMA) flow chart [20]

A total of 449,168 studies were found after searching in PubMed, Google Scholar, and the Cochrane Library. A total of 448,234 were marked as ineligible by an automation tool. There were a total of 934 studies that underwent title and abstract screening, with 874 papers being discarded. The remaining 60 papers were chosen by full-free text evaluation in the previous five years, and after discarding duplicates, resulting in the elimination of 50 studies, only 10 studies were enlisted for the final collection of data (Figure 1). 

After assessing 10 RCTs for quality, we attributed six "+" to two of them and seven "+" to seven. We considered these studies high quality and decided to include them in our systematic review. The results are presented in Table 3:

**Table 3:** Quality assessment of RCTs RCTs, randomized controlled trials

Studies	Random sequence generation (selection bias)	Allocation concealment (selection bias)	Blinding of participants	Blinding of personnel/care providers (performance bias)	Blinding of outcome assessor (detection bias)	Incomplete outcome data (attrition bias)	Selective reporting (reporting bias)	Other biases	Overall
Feng et al. [10]	+	+	+	+	+	+	+	-	7/8
Pohl et al. [11]	+	+	+	+	+	+	+	-	7/8
Amara et al. [12]	+	+	+	+	+	+	+	-	7/8
Yang et al. [13]	+	+	+	+	+	+	+	-	7/8
Kwok et al. [14]	+	+	+	+	+	+	+	-	7/8
Cikajlo et al. [15]	+	+	+	+	?	+	+	-	6/8
Chivers et al. [16]	+	+	+	+	-	+	+	-	7/8
Capecci et al. [17]	+	+	+	+	+	+	+	-	7/8
Calabro et al. [18]	+	+	+	+	?	+	+	-	6/8
Ashburn et al. [19]									

Discussion

In Feng et al.'s research, there was no statistical significance in pre-treatment ratings between the two groups (p > 0.05). Following the intervention, time demonstrated that both groups' Functional Gait Assessment (FGA), Timed Up and Go Test (TUGT), and Berg Balance Scale (BBS) scores were statistically significant (p > 0.05). Nonetheless, when comparing post- and pre-rehabilitation data, the control group did not show any significant differences in the Third Part of Unified Parkinson’s Disease Rating Scale (UPDRS3) scores (p > 0.05). The experimental group outperformed the control group on the TUGT, BBS, FGA, and UPDRS3 (p > 0.05). According to the research, PwP improved their walking and balance after engaging in a 12-week VR training program [10]. Data analysis results revealed that the experimental group improved significantly in the TUGT time, BBS score, FGA score, and UPDRS3 score. When comparing VR therapy to other types of rehabilitation, however, the UPDRS3 score was not statistically significant between the control group's post- and pre-rehabilitation data [10]. Pohl et al. performed another RCT to evaluate the effect of music on PD patients. Based on the findings of the research, participants in the music group reported notable effects, suggesting that they noticed the training benefits of the music-based intervention. The exercise was generally seen as energizing, with the mind becoming more attentive as a result. Positive benefits reported by patients included better posture and dexterity, fewer tremors, higher total bodily coordination, improved mood and cheerfulness, increased endurance, and improved ability to focus, as reported by the patients themselves [11]. The therapists noted changes, such as improved stamina, coordination, attention, and mood. According to this study, applying music intervention in a group environment might possibly improve psychological characteristics, such as alertness, mood, and general quality of life for people with PD. However, the research did not show that the therapy was effective in making improvements in cognitive function, balance, motor-cognitive dual-tasking, or frozen gait compared to the control group [11].

According to Amara's RTC, the research found that those who exercised had significantly better sleep efficiency than people who performed sleep hygiene (p = 0.001) [12]. As a result, they came to the conclusion that the exercise intervention, rather than changes in motor symptoms, was what caused the observed changes in sleep [12]. When compared to a control group that did not exercise and concentrated on sleep hygiene, high-intensity exercise training was shown to have favorable effects on time spent in slow wave sleep, total sleep duration, sleep efficiency, and waking after sleep onset [12]. Because pharmaceutical treatments for sleep problems have limited efficacy and severe side effects, their discovery marks a major development in the quest for non-pharmacological therapy to address this common and debilitating non-motor ailment [12]. Yang et al. performed an RCT to assess the influence of different modalities of gait-task training on the gait-task performance of PwP. They discovered a source with extra information on the subject. According to their findings, cognitive dual-task gait training (CDTT) may be more effective than motor dual-task gait training (MDTT) and control exercise in lowering time during cognitive dual-task walking [13]. Nonetheless, the MDTT was shown to be more effective in lowering gait variability in those with PD than both the control exercise and CDTT. According to the source, we have previously reported significant training-specific benefits of several dual-task gait training approaches in stroke survivors [13]. The benefits of an exercise program have also been seen in PwP. Furthermore, as a consequence of cognitive dual-task training, our subjects improved their motor dual-task walking and single-task walking performance [13]. Kwok et al. discovered that mindfulness yoga for PD (MY-PD) was more helpful than traditional stretching and resistance training exercises in treating symptoms of anxiety and sadness [14]. The MY-PD group showed statistically and clinically substantial improvement in anxiety and depression symptoms [14]. Moreover, the same group showed improvements in movement disorder as measured by Movement Disorder Society Unified Parkinson's Disease Rating Scale scores; the difference in scores between the MY-PD group and the SRTE group was judged clinically inconsequential [14]. In a different study, Cikajlo et al. used the PHANToM haptic device and created special tasks to investigate the effects of dopaminergic treatment. Their results revealed that, although the treatment resulted in considerable improvements in intense movements, it had no effect on the coordinative components of movements [15]. Meanwhile, VR applications need the synchronization of visual and motor control in order to perform accurate motions. A feasible approach would be to combine medicine and frequent physiotherapy with the use of VR technology. This strategy would add another tool to physiotherapy sessions, allowing for more accurate, adaptive, and monitored treatment without putting physiotherapists under undue physical strain [15].

Chivers et al. found that PDSAFE is the largest physiotherapy experiment to reduce falls in PwP. The experiment's 474 randomly allocated participants were much larger than Canning et al.'s previous biggest trial, which included 231 [16]. The researchers were unable to prove that PDSAFE decreased falls in a varied population of PwP. Morris et al. performed an outpatient exercise trial in 2015 [16]. The experiment was reproduced at home with a reduced amount of therapy, but the results were negative [16]. Capecci et al. demonstrated that extensive training using electromechanical devices, such as the G-EO System or a treadmill, may improve gait in mild to advanced PD patients [17,21]. This research found improvements in endurance, gait capacity, performance, daily task independence, and well-being [17,22]. Moreover, this research found that robot-assisted gait training improves performance better than treadmill usage, particularly in those with significant walking handicaps [17,23,24]. Ashburn et al.'s RCT-therapy trial worked. The rehabilitation program was provided by skilled physiotherapists using evidence-based methods. The training program was well structured, and practice sessions were accurately assessed [25]. The average number of supervised sessions was 11 or 12, lasting one to 1.5 hours. The participants were also urged to practice independently every day, as per Sherrington et al.'s studies. The suggested minimum time was 50 hours of practice [19]. The curriculum was rigorous, forcing participants to improve and apply their skills to their daily work. Even if the therapy was tailored to their needs, it may have been difficult for those with severe symptoms [19]. Active engagement and cognitive processing are necessary for PDSAFE and fall prevention self-management [19]. People with considerable cognitive impairment and freezing of gait may have required further supervised fall prevention training [19].

## Conclusions

In this SLR, we take the most valuable outcomes of each of the articles gathered to show an overview of the variety of physical therapies and their adjuvants for the best result in PwP. Our first conclusion obtained about the VR rehabilitation for PwP was statistically significant in TUGT, BBS, and FGA with a p-value of <0.05. The second conclusion was that music as a therapy did not show statistical significance in improving dual tasking, balance, or gait freezing. We state that PwP enhances their sleep after following an exercise program. MDTT showed that it was more effective in reducing the gait difficulties in PwP. MY-PD played an important role in reducing depression and anxiety in PwP. Dopaminergic improvement was seen with the use of PHANToM haptic devices. Devices, such as the G-EO System or a treadmill, resulted in an effective improvement of gait capacity, endurance, and global independence in patients' activities. Thus, more studies for each of the new technological and contemporaneous approaches for a better physical outcome should be conducted for an enhancement of the quality of life of PwP.
